# Vaccine allocation in a declining epidemic

**DOI:** 10.1098/rsif.2012.0404

**Published:** 2012-07-06

**Authors:** E. Goldstein, J. Wallinga, M. Lipsitch

**Affiliations:** 1 Center for Communicable Disease Dynamics, Department of Epidemiology, Harvard School of Public Health, Boston, MA 02115, USA; 2 Department of Immunology and Infectious Diseases, Harvard School of Public Health, Boston, MA 02115, USA; 3 Centre for Infectious Disease Control, National Institute of Public Health and the Environment (RIVM), 3720 BA Bilthoven, The Netherlands

**Keywords:** influenza pandemic, vaccination, high risk, mortality

## Abstract

Sizeable quantities of 2009 pandemic influenza A/H1N1 (H1N1pdm) vaccine in the USA became available at the end of 2009 when the autumn wave of the epidemic was declining. At that point, risk factors for H1N1-related mortality for some of the high-risk groups, particularly adults with underlying health conditions, could be estimated. Although those high-risk groups are natural candidates for being in the top priority tier for vaccine allocation, another candidate group is school-aged children through their role as vectors for transmission affecting the whole community. In this paper, we investigate the question of prioritization for vaccine allocation in a declining epidemic between two groups—a group with a high risk of mortality versus a ‘core’ group with a relatively low risk of mortality but fuelling transmission in the community. We show that epidemic data can be used, under certain assumptions on future decline, seasonality and vaccine efficacy in different population groups, to give a criterion when initial prioritization of a population group with a sufficiently high risk of epidemic-associated mortality is advisable over the policy of prioritizing the core group.

## 1. Introduction

Approximately 62–65% of fatalities among hospitalized H1N1pdm patients were in adults with underlying health conditions other than pregnancy [1,2]. These statistics were obtained during the early stages of an epidemic, when most children were still susceptible to infection; as the epidemic progressed, the proportion of infections among adults (and thus among high-risk adults) would probably increase [3].

Such estimates of the fraction of deaths associated with particular high-risk conditions, combined with estimates of the prevalence of such conditions [1,4–7], permit estimation of the relative risks for mortality among persons with these conditions. Among the groups at the highest risk of dying of pandemic influenza in 2009 were those with renal disease, neurological disorders and perhaps immunosuppression. Providing vaccine to individuals in such groups has a large immediate benefit in preventing mortality, assuming that the vaccine is effective in such groups. On the other hand, vaccination of healthy school-aged children, who are not at high risk of dying, has a benefit in terms of reducing transmission in the community and decreasing the rate of transmission, which would in turn benefit the whole community, including high-risk adults, who form the majority of fatalities. This benefit is particularly pronounced during the early stages of an epidemic; later on its effect is dampened because fewer children are susceptible, hence children play a lesser role in transmission [8]. Prior studies [9,10] have suggested that vaccination of children is optimal, in various senses, when vaccine is available in substantial quantities relatively early in an epidemic, while vaccination of higher risk groups may be more beneficial if vaccine supplies are limited and become available late in an epidemic. These considerations lead to a natural quantitative question: Under what conditions is direct vaccination of the high-risk group members superior as a strategy to vaccination of children to reduce transmission?

The question we consider here is as follows: suppose that small, initial quantities of a vaccine are becoming available during a declining epidemic. Should we give them to the population stratum whose relative risk of fatality is high, or should we give them to the stratum whose relative risk of fatality is low (below average), but which has a strong impact on the epidemic's dynamics in the whole population? The measure of the benefit in both scenarios is the total number of lives that are to be saved throughout the remainder of the epidemic.

We propose to answer this question using a more flexible approach than that adopted in prior studies [9,10]. First, we do not assume knowledge of contact and transmission patterns in different population groups; rather, prioritization is guided by the available data on the epidemic's decline rate. Second, we do not assume that high-risk adults play the same role in the transmission process as adults without underlying medical conditions. Certain high-risk adults may have fewer contacts, particularly with children, and may play a very limited role in transmission to others; in this context, the benefit of allocating vaccine to high-risk adults is only measured using data on their share among the severe outcomes. Third, the approach in both studies [9,10] assumes that a certain vaccine quantity is delivered at once (with no further vaccine distribution) and compares the effect of that allocation for high transmission versus high-risk individuals. In practice, vaccine is produced and distributed gradually during an epidemic. We make no assumption on future vaccine availability in formulating the prioritization criterion. We assume only that the overall transmissibility of the virus does not increase after the point at which the vaccine allocation decision is made; if changing weather or other seasonal factors increase transmission opportunities [11], we assume that such increases are offset by decreasing numbers of susceptible hosts.

Here, we consider this question specifically in the context of a declining epidemic. We note that for the 2009 H1N1 influenza epidemic in the USA, sizeable vaccine quantities became available during the epidemic's declining stage, and, according to recommendations made by the Advisory Committee on Immunization Practices (ACIP) [12], healthy children aged 5–18 and adults with underlying health conditions had the same priority for vaccination. Surveillance data such as those collected by the US Centers for Disease Control and Prevention (CDC) [13] can be used to estimate the weekly decline rate of epidemic incidence. Using this rate and several assumptions, we formulate conditions under which initial prioritization of the high-risk group over the core group is advantageous. We calibrate those conditions against the available data in the USA and assess their relevance for the 2009 H1N1 pandemic, emphasizing several sources of uncertainty, particularly with regard to vaccine efficacy (VE) against fatal outcomes for high-risk adults.

## 2. Method and results

### 2.1. Relative risk between population groups

Let *X* and *Y* be two population subgroups—for example, morbidly obese persons and persons with cancer (note: some individuals may be members of both groups). We define the relative risk *R_t_*(*X*,*Y*) for mortality in group *X* compared with group *Y* at time *t* to be the ratio of the risk of mortality in group *X* at time *t* and the corresponding risk in group *Y*. Here, the risk of mortality in a particular group at time *t* is the number of deaths in that group at time *t* divided by the size of that group.

Data on the prevalence of various underlying conditions among the fatal cases collected during the early stages of an epidemic (e.g. [1,2]), combined with prevalence data for different underlying medical conditions in the population allow for an assessment of *R*_early_(*X*,*W*)—the relative risk of mortality in the various high-risk groups *X* compared with the whole population *W* during the early stages of the epidemic.

We assume that, even as the epidemic progresses, the relative risk for any unvaccinated subgroup of the high-risk group *X* is at least2.1
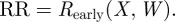
That is, the relative risk of the high-risk group does not decline during the epidemic, relative to the whole population. Although we are not aware of data confirming that assumption, depletion of susceptibles among children and young adults suggests that their relative share among the infected decreases (which could be seen in the decrease of their share among the influenza-like illness (ILI) cases during the H1N1 pandemic [14]), and the share of other population groups (and correspondingly their relative risk of fatality) should increase. The latter increase might not be uniform in all population groups—see more on that in §3.

Table 1 summarizes the prevalence of adults with certain underlying conditions among the fatal H1N1pdm cases in Fowlkes *et al.* [1] and Louie *et al.* [2], and their estimated relative risk of fatality.

**Table 1.** Prevalence among fatal cases from Fowlkes *et al.* [1] and Louie *et al.* [2] and relative risk (RR) for H1N1pdm fatality for certain underlying health conditions in US adults. Ranges are the exact (Clopper and Pearson) confidence intervals for each study. For prevalence of morbid obesity among the fatalities in Fowlkes *et al.* [1], limited body mass index (BMI) data are available, and no absolute counts are reported.

underlying condition (adults)	share among US fatalities	share among US population (%)	RR for fatality
renal disease	12.3% (9–16.4%) [1]	1.288	9.6 (7–12.8) [1]
	15.3% (9.3–23%) [2]		11.9 (7.2–17.9) [2]
neurological disorder/developmental delay	9.9% (6.9–13.7%) [1]	0.91	10.9 (7.5–15) [1]
	11.9% (6.6–19.1%) [2]		13.0 (7.3–21) [2]
immunosuppressive condition	17.6% (13.6–22.2%) [1]	1.9	9.2 (7.2–11.7) [1]
morbid obesity (BMI ≥40)	8.9% [1]	4.47	2.0 [1]
	31.5% (21.2–43.2%) [2]		7.1 (4.8–9.7) [2]
chronic obstructive pulmonary disease	13% (9.5–17.1%) [1]	3.33	3.9 (2.9–5.1) [1]

### 2.2. Effect of vaccination

#### 2.2.1. Targeted vaccination strategies for a limited quantity of vaccines

Suppose that we have enough vaccine to give one dose to a proportion *q* of the whole population—thus, *q* is the number of vaccine doses divided by the number of persons in the whole population *W*. We wish to compare various vaccination strategies for this limited quantity of vaccines and determine for which strategy the number of subsequent deaths is lowest.

Under strategy HR, this vaccine quantity is given to the high-risk group *X*. Under strategy C, this vaccine quantity is given to the core group (children). The only difference between strategy HR and strategy C is in the distribution of this quantity *q*; we assume that the subsequent distribution of vaccine doses beyond *q* is the same in both strategies. A reference strategy, referred to as *N*, leaves this vaccine quantity *q* unused, the subsequent distribution being identical to the HR and C scenarios.

We denote the vaccine efficacy against infection in some population group *G* by 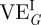, and vaccine efficacy against death by 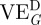. We are mostly interested in estimating the ratio of the vaccine efficacy against death 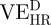 for the high-risk group and the vaccine efficacy against infection 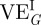 for children:2.2
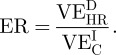
Although we are not aware of data allowing for the estimate of the efficacy ratio (ER), we note that vaccine efficacy against death in any given group might be higher than the efficacy against infection, based on the idea that a limited antibody response to vaccination, insufficient to prevent infection, might still mitigate illness to prevent a fatal outcome.

#### 2.2.2. A comparison result between vaccination strategies

To formulate our main result, we introduce some notation. Let *w*() be the serial interval distribution for influenza (which we assume to be no longer than 7 days), and let *μ* be its mean.

Let *t*_0_ be the day when vaccine quantity *q* takes effect, and *r*_0_ be the epidemic's daily exponential decline rate at that time.

Suppose that distributing quantity *q* to the core group decreases the effective reproductive number of the epidemic by a fraction *Aq*.

Finally, we assume that for the week beyond *t*_0_ the decline rate does not change much under scenario *N*. Under this assumption, we demonstrate (see the electronic supplementary material) that an initial campaign prioritizing the high-risk group over the core group is advantageous in terms of reducing cumulative mortality if2.3
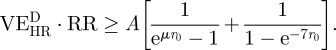


#### 2.2.3. Assessing the prioritization criterion for the 2009 H1N1pdm data in New England

There is some variability in estimates of the serial interval distribution in the literature [15–18]. One can estimate from those papers that the mean *μ* of the serial interval distribution is at least 2.5 days.

To estimate the epidemic's decline rate *r* at different time points, we use surveillance data such as those collected by the CDC [13] following the approach in Goldstein *et al.* [19]. The weekly incidence of influenza 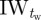 for week *t*_w_ is estimated to be the proportion of ILI among doctor visits multiplied by the proportion of collected specimens testing positive for influenza during that week. The incidence estimate is given up to (an unknown) multiplicative factor; however, the ratio
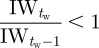
can be thought of as the estimate of the decline rate in incidence during week *t*_w_.

Let the *daily* exponential rate of change in incidence during the week *t*_w_ (assumed constant over that week) be *r*_w_. It is estimated from
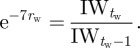


Estimates of the decline rates of the epidemic at the time when sizeable quantities of the vaccine appeared in 2009 varied significantly by different regions in the USA. New England had robust decline rates of the epidemic by the end of November/early December 2009, with the decline subsequently sustained in the winter. Table 2 gives estimates of the daily decline rate between weeks 45 and 48 in New England, on the basis of the ILI and the respiratory specimen testing data collected by the CDC [14].

**Table 2.** Daily exponential decline rate between weeks 45 and 48 in New England.

week	45–46	46–47	47–48
daily decline rate *r*	0.093	0.099	0.12

A method to estimate *A* in equation (2.3) from the epidemic data appears in Wallinga *et al.* [8]. This method suggests that
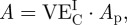
where *A*_p_*q* would be the reduction of the reproductive number under the distribution of quantity *q* of a *perfect* vaccine to children. Equation (2.3) suggests that a criterion for prioritization of a high-risk group with the epidemic's daily decline rate being *r* is2.4
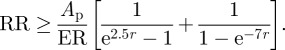


We note that since 0–17 year olds constituted 24.3 per cent of the US population in 2009 [20], and since giving perfect vaccine at random to a fraction *q* of the population reduces the reproductive number by a fraction *q*, one necessarily has that *A*_p_ ≤ 1/0.243 = 4.12 (the latter would be true if other population groups had no impact on the epidemic). Estimation of *A*_p_ based on the method in Wallinga *et al.* [8] applied to the data from the 2009 H1N1 influenza epidemic in the USA is described in the electronic supplementary material. This method suggests a bound2.5
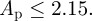


**Figure 1.**
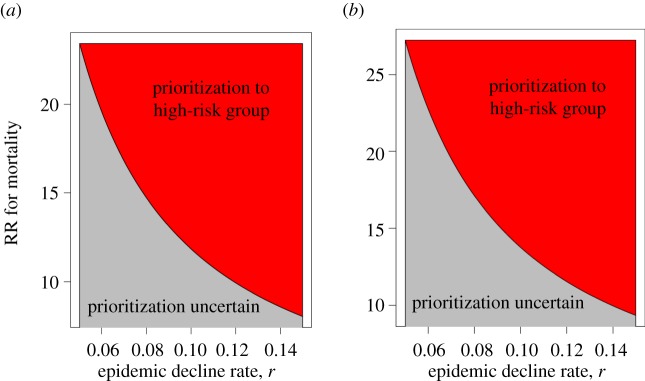
Ranges for the epidemic decline rate *r* and the relative risk (RR) for mortality in a high-risk group (red) for which prioritization of the high-risk group is justifiable under equation (2.4) for different values of the parameter *A*_p_, under the assumption that ER ≥ 1. (*a*) *A*_p_ = 2.15 and (*b*) *A*_p_ = 2.5.

Figure 1 plots a range of values for the decline rate *r* and the relative risk of a fatal outcome, for which our criterion suggests the prioritization of the high-risk group over children for *A*_p_ = 2.15 and 2.5, assuming that ER ≥ 1. We note that for the complementary region, plotted in grey, our criterion is inconclusive, and no prioritization recommendation either for children or for the high-risk adults can be made.

Finally, applying the criterion in equation (2.4) with *A*_p_ ≤ 2.15 (as estimated from the epidemic data in the electronic supplementary material) under the assumption that ER≥1 suggests that, by week 48 of 2009 in New England, prioritization of adults with neuromuscular disorders, renal disease and possibly immunosuppression over healthy children was advisable. On the other hand, if ER is significantly lower than 1, it might have been the case that prioritizing school-aged children was advisable owing to the effect of vaccinating children on the epidemic dynamics and mortality in the whole community. We note that, in New England, adherence to the ACIP guidelines [12] giving equal priority to healthy school-aged children and adults with underlying health conditions was adopted by the local health departments [21,22].

#### 2.2.4. Practical considerations behind prioritization of high-risk individuals

Our proposed strategy is that, when a small quantity of vaccine becomes available during the declining phase of an epidemic, prioritizing high-risk adults can reduce overall mortality compared with prioritizing healthy children, provided the adults in the priority group are at high enough risk, as defined in equation (2.3). When sizeable proportions of the population are already vaccinated, or when the criterion in equation (2.3) is not met, prioritizing high-risk adults might not be warranted, and a larger impact can potentially be obtained by prioritizing children rather than high-risk adults to accelerate the epidemic's decline.

We also note that our approach assumes the feasibility of implementing a flexible vaccine distribution policy. Such a policy would entail a switch from an initial, short-term drive to vaccinate certain groups of high-risk individuals to a campaign for vaccinating children. Prior planning of resources to reach those high-risk individuals, which includes fostering their awareness about the risks they are facing, and the ability to redirect the targeting of the available vaccine on a timely basis are necessary for the approach to be successful.

## 3. Discussion

This paper examines prioritization for vaccine allocation in a declining influenza epidemic. It formulates conditions under which an initial campaign to vaccinate individuals with a high risk of mortality from influenza is preferable to vaccinating a core group, like children, which has a relatively low risk of mortality but fuels transmission in the community. It is shown how those conditions can be validated in real time under a range of uncertainties in the estimates of certain quantities related to the epidemic's progression and VE. We note that, for an emerging epidemic, priority for vaccine allocation with the goal of minimizing the overall mortality burden may go to school-aged children rather than adults with underlying health conditions [9,10].

A basic source of uncertainty in applying the prioritization criterion is the need for an estimate of vaccine efficacy against fatal outcomes in various high-risk groups. Although we are not aware of data assessing the above efficacy, several studies estimating the immunogenicity and efficacy against infection in different high-risk groups for the vaccine against the 2009 A/H1N1 influenza have been published. A case–control study has found poor immune response to vaccination in haemodialysis patients [23]. Some observational data are now available on vaccine efficacy against infection among the high-risk groups for the 2009 H1N1 influenza pandemic [24–26]. These data suggest that vaccine efficacy against infection is lower in high-risk individuals than in healthy children. However, observational studies of influenza vaccine effectiveness may be subject to significant residual confounding, especially among high-risk persons [27]. Moreover, vaccine efficacy against *fatal* outcomes among high-risk adults might be different, either affirming the rationale behind their prioritization as indicated by our approach or suggesting that low efficacy in direct protection of high-risk adults makes prioritization of school-aged children advisable because of the effect of vaccinating children on the epidemic dynamics and mortality in the whole community. Another source of uncertainty related to the impact of vaccination is the potential detrimental short-term effect that vaccination might have on susceptibility. Data from Emborg *et al.* [25] suggest a negative and statistically significant vaccine efficacy against infection occurring within a week from vaccination. Given that, in a declining epidemic, a sizeable fraction of future infections are likely to occur within a fairly short time, such an effect may take away from the benefit of vaccinating high-risk individuals, although it may also have an impact on vaccinating children.

Another potential source of uncertainty in prioritizing high-risk adults is the feasibility of a timely implementation of a vaccination campaign for those groups. For children, rapid administration of a vaccine is possible through school-based vaccination drives. High-risk adults might be a harder target group to reach, with past efforts concentrated on their healthcare providers [28,29]. A combination of risk awareness and a speedy distribution framework is needed to ensure that vaccine allocation to high-risk adults would not lag significantly behind an alternative of administering the corresponding vaccine quantity to children.

Besides vaccine efficacies in different population groups, the key quantities needed to ascertain our prioritization criterion are the epidemic's decline rate and the impact of vaccinating the core group (children) on the epidemic's reproductive number. The decline rate can be estimated from surveillance data, such as those collected by the CDC [13], using a proxy for influenza incidence described in Goldstein *et al.* [19]. The impact of vaccinating the core group on the reproductive number can be gauged following the method in Wallinga *et al.* [8]. That method requires knowledge of the relative susceptibility and infectivity in different population groups, and the epidemic's incidence curve stratified by those groups. Data on relative susceptibility and infectivity in different age groups can be obtained from household studies and may, in principle, become available in real time; we have borrowed the estimates from Cauchemez *et al.* [16], which was published at the end of 2009. We did not have a good estimate of the epidemic's incidence curve; instead, we have adapted the method in Wallinga *et al.* [8] to give an upper bound on the impact of vaccinating children using the final attack rates in different age groups, extrapolated from Zimmer *et al.* [30]. We want to point out that, in principle, it is possible that better surveillance data for the epidemic's progression can produce sharper, real-time estimates of the impact of vaccinating children. Such surveillance data can come from serial serological data [31], or from age-stratified data on ILI and respiratory specimen testing, combined with a real-time serological study, or possibly from syndromic data [32]. The practical aspects of obtaining such data in real time remain uncertain [33].

An assumption we make in our approach is that, as time progresses, depletion of susceptibles owing to natural infections in high-risk groups is slower than the depletion of susceptibles in the whole population (particularly among children and the young adults); thus, the relative risk for the unvaccinated subgroup of a high-risk group increases with time. The role of young individuals during the early stages of an epidemic and their subsequent depletion is known [8], and evidence for the decline of the relative share of the young individuals among the infected can be seen in the ILI data [14]. More evidence for the assumption on the increasing relative risk of severe outcomes in the high-risk groups is provided by Flu.Gov [3]. However, those considerations need not imply that relative risks in each high-risk group increase with time. Children and young adults represent a small fraction of fatal cases, and adults in certain high-risk groups might be more susceptible to infection than other adults in the corresponding age groups, experiencing a larger initial depletion of susceptibles and correspondingly lower relative risk of fatality in later stages of an epidemic. To assess this issue, one can measure the share of individuals with a particular underlying health condition for a certain recorded outcome, e.g. hospitalization, in different age groups. Changes in their relative share through time should give an indication about the change in their relative risk of fatality compared with the early stages of the epidemic.

Yet another assumption in our approach is that the impact of further vaccination and depletion of susceptible individuals is stronger than the impact of seasonality or genetic changes affecting the transmissibility of the virus. For the 2009 H1N1pdm this assumption with regard to seasonality was violated in southeastern USA owing to little willingness in the population to get vaccinated when vaccine was widely available. It is reasonable to assume that for a more pathogenic strain this factor would play a lesser role. At the same time, the decline rate of the epidemic in the southeast was low and a wintertime resurgence owing to seasonal forcing could be predicted [34]. Moreover, under the approach of this paper, such a low decline rate would justify prioritization of risk groups whose relative risks for fatal outcomes are higher than those existing in the published data.

Some regions of the USA, such as New England, had high H1N1pdm vaccination rates and a decline in the epidemic which was sustained through the winter. We assess our criterion for New England and specify the high-risk groups which should have been initially prioritized for vaccination over school-aged children, under certain assumptions on vaccine efficacy against mortality in those high-risk groups. We note that there is a wide range of uncertainty in the available data for the 2009 H1N1 pandemic with regard to the estimates for the relative risk of mortality for individuals with various underlying conditions, as well as for the attack rates and the relative susceptibility and infectivity in the different population groups. More detailed epidemiological data would reduce the above uncertainties, and correspondingly the uncertainty in our conclusions for vaccine prioritization.

## References

[1] Fowlkes A. L. 2011 Epidemiology of 2009 pandemic influenza A (H1N1) deaths in the United States, April–July 2009. Clin. Infect. Dis. 52 (Suppl. 1) S60–S68 10.1093/cid/ciq022 (doi:10.1093/cid/ciq022) 21342901

[2] Louie J. K. 2009 Factors associated with death or hospitalization due to pandemic 2009 influenza A (H1N1) infection in California. J. Am. Med. Assoc. 302, 1896–1902 10.1001/jama.2009.1583 (doi:10.1001/jama.2009.1583) 19887665

[3] Flu.Gov. 2010 U.S. sees increase in H1N1 flu activity, 29 March. See http://www.flu.gov/news/blogs/increasedactivity.html

[4] Pleis J. R. Lucas J. W. Ward B. W. 2009 Summary health statistics for U.S. adults: National health interview survey, 2008. Vital Health Stat. 10, 1–157 20821903

[5] Hirtz D. Thurman D. J. Gwinn-Hardy K. Mohamed M. Chaudhuri A. R. Zalutsky R. 2007 How common are the ‘common’ neurologic disorders? Neurology 68, 326–337 10.1212/01.wnl.0000252807.38124.a3 (doi:10.1212/01.wnl.0000252807.38124.a3) 17261678

[6] Zimmerman R. K. Lauderdale D. S. Tan S. M. Wagener D. K. 2010 Prevalence of high-risk indications for influenza vaccine varies by age, race, and income. Vaccine 28, 6470–6477 10.1016/j.vaccine.2010.07.037 (doi:10.1016/j.vaccine.2010.07.037) 20674882 PMC2939262

[7] Flegal K. M. Carroll M. D. Ogden C. L. Curtin L. R. 2010 Prevalence and trends in obesity among US adults, 1999–2008. J. Am. Med. Assoc. 303, 235–241 10.1001/jama.2009.2014 (doi:10.1001/jama.2009.2014) 20071471

[8] Wallinga J. van Boven M. Lipsitch M. 2010 Optimizing infectious disease interventions during an emerging epidemic. Proc. Natl Acad. Sci. USA 107, 923–928 10.1073/pnas.0908491107 (doi:10.1073/pnas.0908491107) 20080777 PMC2818907

[9] Mylius S. D. Hagenaars T. J. Lugner A. K. Wallinga J. 2008 Optimal allocation of pandemic influenza vaccine depends on age, risk and timing. Vaccine 26, 3742–3749 10.1016/j.vaccine.2008.04.043 (doi:10.1016/j.vaccine.2008.04.043) 18524428

[10] Matrajt L. Jr Longini I. M. 2010 Optimizing vaccine allocation at different points in time during an epidemic. PLoS ONE 5, e13767 10.1371/journal.pone.0013767 (doi:10.1371/journal.pone.0013767) 21085686 PMC2978681

[11] Shaman J. Pitzer V. E. Viboud C. Grenfell B. T. Lipsitch M. 2010 Absolute humidity and the seasonal onset of influenza in the continental United States. PLoS Biol. 8, e1000316 10.1371/journal.pbio.1000316 (doi:10.1371/journal.pbio.1000316) 20186267 PMC2826374

[12] US Centers for Disease Control and Prevention. 2009 ACIP vaccination recommendations for the 2009 H1N1 influenza epidemic See http://www.cdc.gov/media/pressrel/2009/r090729b.htm

[13] US Centers for Disease Control and Prevention. FluView, US CDC Influenza Division See http://www.cdc.gov/flu/weekly/

[14] US Centers for Disease Control and Prevention. United States surveillance data See http://www.cdc.gov/flu/weekly/ussurvdata.htm

[15] Donnelly C. A. 2011 Serial intervals and the temporal distribution of secondary infections within households of 2009 pandemic influenza A (H1N1): implications for influenza control recommendations. Clin. Infect. Dis. 52 (Suppl. 1), S123–S130 10.1093/cid/ciq028 (doi:10.1093/cid/ciq028) 21342883 PMC3106264

[16] Cauchemez S. Donnelly C. A. Reed C. Ghani A. C. Fraser C. Kent C. K. Finelli L. Ferguson N. M. 2009 Household transmission of 2009 pandemic influenza A (H1N1) virus in the United States. N. Engl. J. Med. 361, 2619–2627 10.1056/NEJMoa0905498 (doi:10.1056/NEJMoa0905498) 20042753 PMC3840270

[17] Yang Y. Sugimoto J. D. Halloran M. E. Basta N. E. Chao D. L. Matrajt L. Potter G. Kenah E. Longini I. M. 2009 The transmissibility and control of pandemic influenza A (H1N1) virus. Science 326, 729–733 10.1126/science.1177373 (doi:10.1126/science.1177373) 19745114 PMC2880578

[18] Cowling B. J. Fang V. J. Riley S. Malik Peiris J. S. Leung G. M. 2009 Estimation of the serial interval of influenza. Epidemiology 20, 344–347 10.1097/EDE.0b013e31819d1092 (doi:10.1097/EDE.0b013e31819d1092) 19279492 PMC3057478

[19] Goldstein E. Cobey S. Takahashi S. Miller J. Lipsitch M. 2011 Predicting the epidemic sizes of influenza A/H1N1, A/H3N2 and B: a statistical method. PLoS Med. 8, e1001051 10.1371/journal.pmed.1001051 (doi:10.1371/journal.pmed.1001051) 21750666 PMC3130020

[20] US Centers for Disease Control and Prevention. United States census estimates See http://wonder.cdc.gov/bridged-race-population.html

[21] Massachusetts Department of Public Health. 2009 Recommendations for the use of influenza A (H1N1) 2009 *monovalent vaccine*. See http://www.neias.org/pdf/MA09VInactH1N1OrdersRev112309.pdf

[22] Connecticut Department of Public Health. H1N1 vaccine distribution response plan See http://www.ct.gov/ctfluwatch/lib/ctfluwatch/h1n1/h1n1_vaccine_dist_plan_809.pdf

[23] Chang Y. T. Guo C. Y. Tsai M. S. Cheng Y. Y. Lin M. T. Chen C. H. Shen D. Wang J. R. Sung J. M. 2012 Poor immune response to a standard single dose non-adjuvenated vaccination against 2009 pandemic H1N1 influenza virus A in the adult and elder hemodialysis patients. Vaccine 30, 5009–5018 10.1016/j.vaccine.2012.05.016 (doi:10.1016/j.vaccine.2012.05.016) 22658967

[24] Andrews N. Waight P. Yung C. F. Miller E. 2011 Age-specific effectiveness of an oil-in-water adjuvanted pandemic (H1N1) 2009 vaccine against confirmed infection in high risk groups in England. J. Infect. Dis. 203, 32–39 10.1093/infdis/jiq014 (doi:10.1093/infdis/jiq014) 21148494 PMC3086445

[25] Emborg H. D. Krause T. G. Hviid A. Simonsen J. Molbak K. 2012 Effectiveness of vaccine against pandemic influenza A/H1N1 among people with underlying chronic diseases: cohort study, Denmark, 2009–10. Br. Med. J. 344, d7901 10.1136/bmj.d7901 (doi:10.1136/bmj.d7901) 22277542

[26] Simpson C. R. Ritchie L. D. Robertson C. Sheikh A. McMenamin J. 2010 Vaccine effectiveness in pandemic influenza—primary care reporting (VIPER): an observational study to assess the effectiveness of the pandemic influenza A (H1N1)v vaccine. Health Technol. Assess. 14, 313–346 20630126 10.3310/hta14340-05

[27] Simonsen L. Taylor R. J. Viboud C. Miller M. A. Jackson L. A. 2007 Mortality benefits of influenza vaccination in elderly people: an ongoing controversy. Lancet Infect. Dis. 7, 658–666 10.1016/S1473-3099(07)70236-0 (doi:10.1016/S1473-3099(07)70236-0) 17897608

[28] UK Health Protection Agency. Swine flu vaccination: what you need to know See http://www.direct.gov.uk/prod_consum_dg/groups/dg_digitalassets/@dg/@en/documents/digitalasset/dg_181508.pdf

[29] Kettler B. 2009 Focus of H1N1 vaccination effort shifts. *Mail Tribune*, 14 November 2009. See http://www.mailtribune.com/apps/pbcs.dll/article?AID=/20091114/NEWS/911140310

[30] Zimmer S. 2010 Seroprevalence following the second wave of pandemic 2009 H1N1 influenza in Pittsburgh, PA, USA. PLoS ONE 5, e11601 10.1371/journal.pone.0011601 (doi:10.1371/journal.pone.0011601) 20644650 PMC2904390

[31] Wu J. T. 2011 Estimating infection attack rates and severity in real time during an influenza pandemic: analysis of serial cross-sectional serologic surveillance data. PLoS Med. 8, e1001103 10.1371/journal.pmed.1001103 (doi:10.1371/journal.pmed.1001103) 21990967 PMC3186812

[32] Goldstein E. Cowling B. J. Aiello A. E. Takahashi S. King G. Lu Y. Lipsitch M. Yates A. 2011 Estimating incidence curves of several infections using symptom surveillance data. PLoS ONE 6, e23380 10.1371/journal.pone.0023380 (doi:10.1371/journal.pone.0023380) 21887246 PMC3160845

[33] Lipsitch M. Finelli L. Heffernan R. T. Leung G. M. Redd S. C. 2011 Improving the evidence base for decision making during a pandemic: the example of 2009 influenza A/H1N1. Biosecur. Bioterror. 9, 89–115 21612363 10.1089/bsp.2011.0007 PMC3102310

[34] Shaman J. Goldstein E. Lipsitch M. 2011 Absolute humidity and pandemic versus epidemic influenza. Am. J. Epidemiol. 173, 127–135 10.1093/aje/kwq347 (doi:10.1093/aje/kwq347) 21081646 PMC3011950

